# Non‐Markov Nonparametric Estimation of Complex Multistate Outcomes After Hematopoietic Stem Cell Transplantation

**DOI:** 10.1002/bimj.70082

**Published:** 2025-10-29

**Authors:** Judith Vilsmeier, Sandra Schmeller, Daniel Fürst, Jan Beyersmann

**Affiliations:** ^1^ Institute of Statistics Ulm University Ulm Germany; ^2^ Institute for Clinical Transfusion Medicine and Immunogenetics Ulm German Red Cross Blood Transfusion Service Baden Wuerttemberg ‐ Hessen and University Clinic Ulm Ulm Germany; ^3^ Institute of Transfusion Medicine Ulm University Ulm Germany

**Keywords:** multistate models, non‐Markov, recurrent events, wild bootstrap

## Abstract

Often probabilities of nonstandard time‐to‐event endpoints are of interest, which are more complex than overall survival. One such probability is chronic graft‐versus‐host disease (GvHD‐) and relapse‐free survival, the probability of being alive, in remission, and not suffering from chronic GvHD after stem cell transplantation, with chronic GvHD being a recurrent event. Because the probabilities for endpoints with recurrent events may not fall monotonically, one should not use the Kaplan–Meier estimator for estimation, but the Aalen–Johansen estimator. The Aalen–Johansen is a consistent estimator even in non‐Markov scenarios if state occupation probabilities are being estimated and censoring is random. In some multistate models, it is also possible to use linear combinations of Kaplan–Meier estimators, which do not depend on the Markov assumption but can estimate probabilities to be out of bounds. For these linear combinations, we propose a wild bootstrap procedure for inference and compare it with the wild bootstrap for the Aalen–Johansen estimator in non‐Markov scenarios. In the proposed procedure, the limiting distribution of the Nelson–Aalen estimator is approximated using the wild bootstrap and transformed via the functional delta method. This approach is adaptable to different multistate models. Using real data, confidence bands are generated using the wild bootstrap for chronic GvHD‐ and relapse‐free survival. Additionally, coverage probabilities of confidence intervals and confidence bands generated by Efron's bootstrap and the wild bootstrap are examined with simulations.

## 1 Introduction

The analysis of complex nonstandard endpoints is an important topic in many research areas, as it allows to combine multiple endpoints, to investigate more complex scientific questions, and to incorporate recurrent events. An example of this is the assessment of the success of hematopoietic stem cell transplantation, where it may be appropriate to consider not only death and relapse but also the occurrence of graft‐versus‐host disease (GvHD), as it can impair the quality of life of those affected. Here, GvHD is considered as a recurrent event as this disease can reappear several times. The inclusion of GvHD as a recurrent event in the assessment of hematopoietic stem cell transplantation is also reflected in the literature, in which endpoints are investigated that include GvHD or the use of immunosuppressants as recurrent events (Klein et al. 2000; Bluhmki et al. 2020; Solomon et al. 2017). The focus on the estimation of probabilities corresponds to current guidelines to rather analyze risks and not rates, especially if more than one hazard plays a role for the probability of interest (Andersen et al. 2021; Bühler et al. 2023).

**FIGURE 1:**
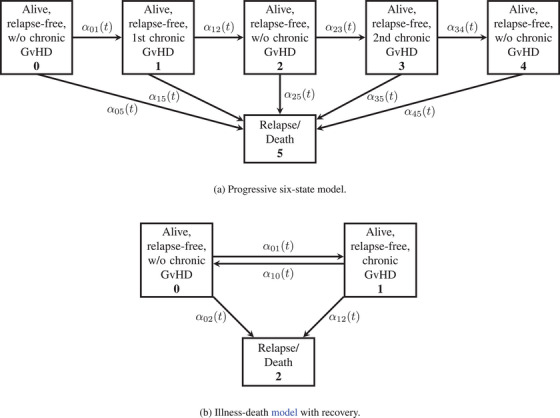
Two models with chronic GvHD as a recurrent event.

When analyzing endpoints with recurrent events, it is important to note that time‐to‐first‐event analyses do not capture the entire course of the disease as they ignore information about recurrent events. Additionally, one should not use a simple Kaplan–Meier estimator for the estimation of the probability of being event‐free as this probability may not fall monotonically but may also rise again, if recurrent events are involved. Instead, multistate models can be used to describe the course of the disease and the Aalen–Johansen estimator can be used to estimate probabilities of complex endpoints. Two multistate models, which were used in recent literature to analyze complex endpoints in the context of stem cell transplantation, are the illness–death model with recovery (Bluhmki et al. 2020) and the progressive six‐state model (Solomon et al. 2017; Zhang et al. 2022). The illness–death model (Figure 1b) is a suitable model if few data are available for estimation as in this model all patients who are alive and in remission are aggregated in only two states and therefore fewer quantities are being estimated, but the fulfillment of the Markov assumption is often questionable. In the progressive six‐state model (Figure 1a), fewer data are available for estimation than in the illness–death model with recovery. However, the Markov assumption is more plausible in progressive models, which may lead to a preference for these models over the illness–death model with recovery. Furthermore, there are no back transitions in progressive models so they allow for the use of linear combinations of Kaplan–Meier estimators (Pepe 1991), which do not depend on the Markov assumption for consistency. This was seen as an advantage, as in the past, the Aalen–Johansen estimator was assumed to be consistent only if the time‐inhomogeneous Markov assumption is fulfilled.

But the Aalen–Johansen estimator consistently estimates state occupation probabilities even in the non‐Markov case, as long as censoring is random (Datta and Satten 2001; Overgaard 2019; Nießl et al. 2023), so the illness–death model with recovery is again of interest for estimating complex outcomes with recurrent events. Practical experience has shown that the Aalen–Johansen estimator and linear combinations of Kaplan–Meier estimators give similar results when estimating state occupation probabilities in non‐Markov models (Andersen and Pohar Perme 2008; Klein et al. 2000; Meira‐Machado and Sestelo 2019). But only few simulations of non‐Markov models were conducted, and those were mostly limited to the simpler illness–death model without recovery. Therefore, we examine how the Aalen–Johansen estimator in the illness–death model with recovery performs in non‐Markov settings compared to the Aalen–Johansen estimator in the progressive six‐state model and the linear combination of Kaplan–Meier estimators, which is independent of the fulfillment of the Markov assumption. The quantity being estimated for the comparison is the probability that a patient is still alive and in remission at a time t after transplantation and is not suffering from chronic GvHD, in the following referred to as chronic GvHD‐ and relapse‐free survival (CGRFS), with chronic GvHD being a recurrent event.

Additionally, we investigate differences between confidence bands, as well as confidence intervals, for chronic GvHD‐ and relapse‐free survival estimated with the three estimators of interest. To obtain the confidence bands and intervals, two different bootstrap methods are used: the first being Efron's bootstrap, which samples units with replacement, and the second being the wild bootstrap. The wild bootstrap resamples martingale errors and is an interesting alternative to Efron's bootstrap when dealing with survival data, as it provides reliable results not only if censoring is random, but also with more general censoring schemes if the Markov assumption is fulfilled (Nießl et al. 2023; Rühl et al. 2023).

For this purpose, a wild bootstrap approach for linear combinations of Kaplan–Meier estimations is derived, by approximating the limiting distribution of the Nelson–Aalen estimator with the wild bootstrap and transforming the results using a functional delta method argument. The approach is inspired by Bluhmki et al. (2018), who derived a wild bootstrapping procedure along these lines for the general Aalen–Johansen estimator under a Markov assumption, but not for subsequent functionals. The idea will be to transform according to a subsequent Hadamard derivative for a functional such as chronic GvHD‐ and relapse‐free survival and use the observation of Nießl et al. (2023) that the major variation of the estimator is captured by the Doob–Meyer decomposition of the individual transition‐specific counting processes even in non‐Markov models. Our proposal includes the special and simpler case of Liu et al. (2008) and justifies their procedure. Furthermore, our approach can be easily adapted to other multistate models and linear combinations of Kaplan–Meier estimators, although it was derived here using the progressive six‐state model as an example.

Sections 2 and 3 give an overview of multistate models in general and the models and estimators used to estimate chronic GvHD‐ and relapse‐free survival. In Section 4, we use a toy example to demonstrate that linear combinations of Kaplan–Meier estimators can estimate probabilities to be out of bounds. The wild bootstrap approach for linear combinations of Kaplan–Meier estimations is derived in Section 5. In Section 6, log–log transformed confidence bands and confidence intervals are constructed using Efron's bootstrap and the wild bootstrap. In Section 7, chronic GvHD‐ and relapse‐free survival is estimated with all three estimators using real data from the German Registry for Hematopoietic Stem Cell Transplantation and Cell Therapy, for which tests indicate that the Markov assumption is not fulfilled. Additionally, confidence bands are obtained using the wild bootstrap. Moreover, in Section 8, the coverage probabilities of confidence intervals and confidence bands generated by Efron's bootstrap and by the wild bootstrap are analyzed in simulations in which the Markov property is deliberately violated.

## 2 Multistate Models

Let [0,∞)∋t↦X(t) be a multistate stochastic process, which is càdlàg and has a finite state space G={0,⋯,K−1}. X(t) indicates which state in the multistate model is occupied at time t. We allow X(t) to be non‐Markov.

In multistate models, the probability that an individual is in state h at time t, given that the individual was in state g at time s, is the transition probability

$$P_{gh}\left(s,t\right):=P\left(X\left(t\right)=h\;|\;X\left(s\right)=g\right),\;g,h\in G,\;s\leq t.$$

Other probabilities that may be of interest are the state occupation probabilities

P(X(t)=h),h∈G.

In the case that all individuals have the same initial state g, that is, P(X(0)=g)=1, the state occupation probabilities correspond to the transition probabilities $P_{gh}\left(0,t\right)$.

The partly conditional transition rate of a g→h transition is defined via

$$\alpha_{gh}\left(t\right)\cdot dt:=P\left(X\left(t+dt\right)=h\;|\;X\left(t-\right)=g\right),\;g,h\in G,\;g\neq h,$$

assuming the limits to exist. The rate $\alpha_{gh}\left(t\right)$ corresponds to the instantaneous risk of an individual who is in state g to transition to state h. Note that (4) only conditions on the immediate past at t−, but not on the entire past before t, and that a Markov assumption is not made.

Based on the partly conditional transition rates, the cumulative transition rate is defined as

$$A_{gh}\left(t\right)=\int_{0}^{t}\alpha_{gh}\left(u\right)du,$$

which is continuous. Accordingly, the matrix of cumulative transition rates A(t) is defined as ${\left\{A\left(t\right)\right\}}_{gh}=A_{gh}\left(t\right)$ for g≠h and ${\left\{A\left(t\right)\right\}}_{gg}=-\sum_{h=0,h\neq g}^{K-1}A_{gh}\left(t\right),\;g=0,\cdots ,K-1$, so that the row sums are zero.

The observation of X is subject to right‐censoring by C. Here, it is important to distinguish between random censoring and independent censoring. For this, consider independently and identically distributed data of n individuals, ${\left(X_{i}\left(t\right)\right)}_{t\in (0,\min\left(C_{i},T_{i}\right)]}$, i=1,⋯,n, with $T_{i}$ the time until absorption of individual i and $C_{i}$ its right‐censoring time.

Let $Y_{g}\left(t\right)=\sum_{i=1}^{n}Y_{i;g}\left(t\right)$ be the number of individuals at risk in state g just before t, where $Y_{i;g}\left(t\right)=1\left\{X_{i}\left(t-\right)=g,\;t\leq C_{i}\right\}$ is the individual at‐risk process. Additionally, let $N_{gh}\left(t\right)=\sum_{i=1}^{n}N_{i;gh}\left(t\right)$ be a counting process, which counts the number of observed g→h transitions in the time interval [0,t] over all individuals, with $N_{i;gh}\left(t\right)$ counting the observed g→h transitions in the time interval [0,t] of individual i.

With these definitions, right‐censoring is independent if the censoring preserves the form of the intensity processes of these counting processes (see Andersen et al. 1993, Aalen et al. 2008 for details). This is an extension to the concept of random censoring, where it is assumed that the multistate process X and, hence, time until absorption T are stochastically independent of the right‐censoring time C.

## 3 Estimation of Chronic GvHD‐ and Relapse‐Free Survival

For the analysis of chronic GvHD‐ and relapse‐free survival, two different models are used, whereby in one model the probabilities are estimated with a linear combination of Kaplan–Meier estimators and with the Aalen–Johansen estimator and in the other model only with the Aalen–Johansen estimator. The first model was used by Solomon et al. (2017) and is shown in Figure 1a. In this model, the course of the disease is represented as a progressive chain of events. For this reason, it is referred to in the following as the progressive six‐state model. The second model is based on the model used by Bluhmki et al. (2020) and can be seen in Figure 1b. This model is referred to as the illness–death model with recovery.

In the progressive six‐state model, chronic GvHD‐ and relapse‐free survival corresponds to the sum of the state occupation probabilities P(X(t)=0), P(X(t)=2), and P(X(t)=4). Given that all individuals are alive at the beginning of the observation period, have not had a relapse, and do not suffer from chronic GvHD, it follows that all individuals have the same initial state 0. Thus, the state occupation probabilities P(X(t)=h) correspond to the transition probabilities $P_{0h}\left(0,t\right)$, h∈{0,⋯,5}.

In this model, it is possible to estimate these probabilities with a linear combination of five Kaplan–Meier estimators, which are defined by Solomon et al. (2017) as follows:

${\hat{S}}_{1}$
Events for the first Kaplan–Meier estimator are the beginning of the first episode of chronic GvHD, as well as death or relapse before the first episode of chronic GvHD.
${\hat{S}}_{2}$
For the second Kaplan–Meier estimator, events are the beginning of the second episode of chronic GvHD, as well as death or relapse before the second episode of chronic GvHD.
${\hat{S}}_{3}$
Events for the third Kaplan–Meier estimator are recovery from the first episode of chronic GvHD, as well as death or relapse before recovery from the first episode of chronic GvHD.
${\hat{S}}_{4}$
The fourth Kaplan–Meier estimator corresponds to relapse‐free survival. Thus, any death or relapse is an event.
${\hat{S}}_{5}$
For the fifth Kaplan–Meier estimator, events are recovery from the second episode of chronic GvHD, as well as death or relapse before recovery from the second episode of chronic GvHD.

${\hat{S}}_{1}\left(t\right)$ estimates the probability of being in state 0 at time t, that is, $P_{00}\left(0,t\right)$. The transition probability $P_{02}\left(0,t\right)$ can be estimated by ${\hat{S}}_{2}\left(t\right)-{\hat{S}}_{3}\left(t\right)$ and ${\hat{S}}_{4}\left(t\right)-{\hat{S}}_{5}\left(t\right)$ estimates $P_{04}\left(0,t\right)$.

Thus, chronic GvHD‐ and relapse‐free survival can be estimated as

$$\hat{\text{CGRFS}}\left(t\right)={\hat{S}}_{1}\left(t\right)+{\hat{S}}_{2}\left(t\right)-{\hat{S}}_{3}\left(t\right)+{\hat{S}}_{4}\left(t\right)-{\hat{S}}_{5}\left(t\right).$$

To express the Kaplan–Meier estimators in terms of multistate models, define the Nelson–Aalen estimator as

$${\hat{A}}_{gh}\left(t\right)=\int_{0}^{t}\frac{1\{Y_{g}\left(u\right)>0\}}{Y_{g}\left(u\right)}dN_{gh}\left(u\right).$$

The Nelson–Aalen estimator is a consistent estimator for the cumulative rate $A_{gh}\left(t\right)$ even in the non‐Markov case, as long as censoring is random (Nießl et al. 2023).

With this definition and the regularity assumption $\sup_{s\in [0,\tau ]}\left|n^{-1}Y_{g}\left(s\right)-y_{g}\left(s\right)\right|\overset{P}{\to }0\;\forall g\in G$ as n→∞, where $y_{g}:\left[0,\tau \right]\to \left(0,1\right]$ are deterministic, positive functions, the five Kaplan–Meier estimators ${\hat{S}}_{j}$ can be expressed as

$${\hat{S}}_{j}\left(t\right)=S\left\{{\hat{f}}_{j}\left(\hat{A}\right)\right\}\left(t\right),\;j=1,\cdots ,5,$$

with 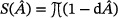, where 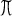 denotes the product integral and $\hat{A}\left(t\right)$ a matrix with non‐diagonal entries ${\hat{A}}_{gh}\left(t\right)$ and diagonal entries such that the row sums are zero. The functions ${\hat{f}}_{1}\left(\hat{A}\right),\cdots ,{\hat{f}}_{5}\left(\hat{A}\right)$ are obtained by replacing $y_{g}$ with the at‐risk process $Y_{g}$ in the functions $f_{1}\left(\hat{A}\right),\cdots ,f_{5}\left(\hat{A}\right)$, which are given by

$$\begin{array}{cc} f_{1}\left(\hat{A}\right) & ={\hat{A}}_{01}+{\hat{A}}_{05},\\ f_{2}\left(\hat{A}\right) & =\frac{y_{0}{\hat{A}}_{05}}{y_{0}+y_{1}+y_{2}}+\frac{y_{1}{\hat{A}}_{15}}{y_{0}+y_{1}+y_{2}}+\frac{y_{2}\left({\hat{A}}_{25}+{\hat{A}}_{23}\right)}{y_{0}+y_{1}+y_{2}},\\ f_{3}\left(\hat{A}\right) & =\frac{y_{0}{\hat{A}}_{05}}{y_{0}+y_{1}}+\frac{y_{1}\left({\hat{A}}_{15}+{\hat{A}}_{12}\right)}{y_{0}+y_{1}},\\ f_{4}\left(\hat{A}\right) & =\frac{y_{0}{\hat{A}}_{05}}{y_{0}+y_{1}+y_{2}+y_{3}+y_{4}}+\frac{y_{1}{\hat{A}}_{15}}{y_{0}+y_{1}+y_{2}+y_{3}+y_{4}}\\ & \;+\frac{y_{2}{\hat{A}}_{25}}{y_{0}+y_{1}+y_{2}+y_{3}+y_{4}}+\frac{y_{3}{\hat{A}}_{35}}{y_{0}+y_{1}+y_{2}+y_{3}+y_{4}}\\ & \;+\frac{y_{4}{\hat{A}}_{45}}{y_{0}+y_{1}+y_{2}+y_{3}+y_{4}},\\ f_{5}\left(\hat{A}\right) & =\frac{y_{0}{\hat{A}}_{05}}{y_{0}+y_{1}+y_{2}+y_{3}}+\frac{y_{1}{\hat{A}}_{15}}{y_{0}+y_{1}+y_{2}+y_{3}}\\ & \;+\frac{y_{2}{\hat{A}}_{25}}{y_{0}+y_{1}+y_{2}+y_{3}}+\frac{y_{3}\left({\hat{A}}_{35}+{\hat{A}}_{34}\right)}{y_{0}+y_{1}+y_{2}+y_{3}}. \end{array}$$

The functions $f_{j}\left(\hat{A}\right)$ are linear transformations, since it holds that $f_{j}\left(\hat{A}\right)\left(t\right)=u_{j}{\left(t\right)}^{T}\cdot \hat{A}\left(t\right)\cdot v_{j}\left(t\right),\;j=1,\cdots ,5$, with $u_{j}\left(t\right)$ and $v_{j}\left(t\right)$ chosen such that the equations above are obtained.

It also holds that

$$\begin{array}{c} \sqrt{n}\left[{\hat{S}}_{j}\left(t\right)-S\left\{f_{j}\left(\hat{A}\right)\right\}\left(t\right)\right]\overset{P}{\to }0,\;j=1,\cdots ,5. \end{array}$$

Using ${\hat{S}}_{3}$ as an example, this means that it holds that


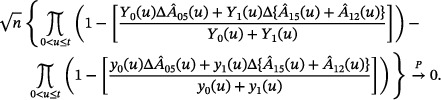

To prove this convergence, it suffices to show that

(1) $$\sqrt{n}\int_{0}^{t}\left\{\frac{Y_{0}\left(u\right)}{Y_{0}\left(u\right)+Y_{1}\left(u\right)}-\frac{y_{0}\left(u\right)}{y_{0}\left(u\right)+y_{1}\left(u\right)}\right\}d{\hat{A}}_{05}\left(u\right)\overset{P}{\to }0$$

and

(2) $$\sqrt{n}\int_{0}^{t}\left\{\frac{Y_{1}\left(u\right)}{Y_{0}\left(u\right)+Y_{1}\left(u\right)}-\frac{y_{1}\left(u\right)}{y_{0}\left(u\right)+y_{1}\left(u\right)}\right\}d\left({\hat{A}}_{15}+{\hat{A}}_{12}\right)\left(u\right)\overset{P}{\to }0.\;\;\;$$

Here, we will argue heuristically that (1), and analogously (2), holds. A formal proof can be found in the Appendix.

If one has a look at Equation (1), one can see that it is equal to

$$\int_{0}^{t}\sqrt{n}\left\{\frac{Y_{0}\left(u\right)/n}{Y_{0}\left(u\right)/n+Y_{1}\left(u\right)/n}-\frac{y_{0}\left(u\right)}{y_{0}\left(u\right)+y_{1}\left(u\right)}\right\}\cdot \frac{1}{n}\cdot \frac{dN_{05}\left(u\right)}{Y_{0}\left(u\right)/n}.$$

The first part of the integrand, $\sqrt{n}\left[\left\{Y_{0}\left(u\right)/n\right\}/\left\{Y_{0}\left(u\right)/n+Y_{1}\left(u\right)/n\right\}-y_{0}\left(u\right)/\left\{y_{0}\left(u\right)+y_{1}\left(u\right)\right\}\right]$, is asymptotically a random variable with expectation zero. Additionally, it holds that $nY_{0}^{-1}\left(u\right)$ is bound away from zero and asymptotically equal to $y_{0}^{-1}\left(u\right)$. Therefore, one can argue heuristically that

$$\int_{0}^{t}\sqrt{n}\left\{\frac{Y_{0}\left(u\right)/n}{Y_{0}\left(u\right)/n+Y_{1}\left(u\right)/n}-\frac{y_{0}\left(u\right)}{y_{0}\left(u\right)+y_{1}\left(u\right)}\right\}\cdot \frac{1}{n}\cdot \frac{dN_{05}\left(u\right)}{Y_{0}\left(u\right)/n}\overset{P}{\to }0,$$

using the weak law of large numbers.

An alternative to the linear combination of Kaplan–Meier estimators is the Aalen–Johansen estimator. This estimator is defined as

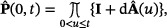

where $\hat{P}\left(0,t\right)$ is the matrix of estimated transition probabilities with ${\left\{\hat{P}\left(0,t\right)\right\}}_{gh}={\hat{P}}_{gh}\left(0,t\right)$ and I is the identity matrix. The Aalen–Johansen estimator is used to directly estimate the transition probabilities ${\hat{P}}_{00}\left(0,t\right)$, ${\hat{P}}_{02}\left(0,t\right)$, and ${\hat{P}}_{04}\left(0,t\right)$ in the progressive six‐state model. So, to summarize, in the progressive six‐state model, there are two estimators available to estimate chronic GvHD‐ and relapse‐free survival: the linear combination of Kaplan–Meier estimators and the Aalen–Johansen estimator. Both will be evaluated in the later sections.

In the illness–death model with recovery, chronic GvHD‐ and relapse‐free survival corresponds to the state occupation probability P(X(t)=0), which is equal to the transition probability $P_{00}\left(0,t\right)$, since all individuals have the same initial state 0. In this model, only the Aalen–Johansen estimator is used to estimate the transition probabilities. Using a linear combination of Kaplan–Meier estimators is not possible, due to the possible back transitions.

The fact that the underlying stochastic process is allowed to be non‐Markov does not pose a problem for the consistency of the Aalen–Johansen estimator, as long as censoring is random and state occupation probabilities are being estimated (Datta and Satten 2001; Overgaard 2019; Nießl et al. 2023). The latter is fulfilled due to the design of the two models; therefore, only the random censoring is a requirement for the eventual study design.

## 4 Exceeding Limits of the Linear Combination of Kaplan–Meier Estimators

It is possible that the chronic GvHD‐ and relapse‐free survival is estimated to be negative or greater than one when using the linear combination of Kaplan–Meier estimators as described in Section 3. This is demonstrated using the following illustrative example with only three individuals shown in Table 1. The first individual is censored before any transition can be observed. Individual 2 has two episodes of chronic GvHD and dies before recovery from the second episode. The third individual experiences one episode of chronic GvHD and is censored before a recovery can be observed.

**TABLE 1:** Example data with three individuals.

ID	From	To	Time
1	0	“cens”	2
2	0	1	1
2	1	2	3
2	2	3	6
2	3	5	7
3	0	1	4
3	1	“cens”	5

Table 2 shows the results of the respective estimators for the time points from 0 to 7. Chronic GvHD‐ and relapse‐free survival is calculated as described in Section 3. At time t=3, the chronic GvHD‐ and relapse‐free survival is estimated to be greater than one. From time t=6, the estimated chronic GvHD‐ and relapse‐free survival becomes negative, since ${\hat{S}}_{3}$ is larger than ${\hat{S}}_{2}$ from this time point on.

**TABLE 2:** Results of the respective Kaplan–Meier estimators and the estimator for chronic GvHD‐ and relapse‐free survival on the example data in Table 1.

t	${\hat{S}}_{1}\left(t\right)$	${\hat{S}}_{2}\left(t\right)$	${\hat{S}}_{3}\left(t\right)$	${\hat{S}}_{4}\left(t\right)$	${\hat{S}}_{5}\left(t\right)$	$\hat{\text{CGRFS}}\left(t\right)$
0	1	1	1	1	1	1
1	0.67	1	1	1	1	0.67
2	0.67	1	1	1	1	0.67
3	0.67	1	0.5	1	1	1.17
4	0	1	0.5	1	1	0.5
5	0	1	0.5	1	1	0.5
6	0	0	0.5	1	1	−0.5
7	0	0	0.5	0	0	−0.5

While in this section only a small toy data example was used for demonstration, this phenomenon can also be observed in larger datasets. In 187 of the 1000 simulated datasets with 200 patients in Section 8, chronic GvHD‐ and relapse‐free survival was estimated to be negative by the linear combination of Kaplan–Meier estimators and in one dataset chronic GvHD‐ and relapse‐free survival was estimated to be greater than one. In such cases, it is necessary to consider how to deal with values that exceed the limits. A straightforward approach is to set all negative values to zero and values greater than one to one, which adds some bias, but ensures that the estimated probabilities stay within the interval [0,1].

## 5 Wild Bootstrap for Linear Combinations of Kaplan–Meier Estimators

The proposed wild bootstrap replaces individual, transition‐specific martingale increments with their corresponding counting process increments, treated as fixed given the data, times independent random variables with mean zero and variance one, a standard choice being standard normal variates. In a non‐Markov model, this approach is still available on the individual, transition‐specific level, but Nelson–Aalen estimation now targets a partly conditional cumulative transition rate, where the average of individual transition intensities approaches the partly conditional rate provided that censoring is entirely unrelated, that is, random. The fact that the individual transition intensities depend on the past if the Markov assumption is not fulfilled introduces extra randomness, but Nießl et al. (2023) suggested that the major variation is in the martingale increments. Hence, we proceed by calculating Hadamard derivatives that will be used to transform martingale representations and weak convergence results under a Markov assumption. A special and simpler case is Liu et al. (2008), who considered Kaplan–Meier combinations in a non‐Markov four‐state model and replaced unknown non‐Markov intensities by Nelson–Aalen increments in representations specific to their model. We will investigate the proposal in non‐Markov settings in Sections 7 and 8.

Under usual regularity assumptions, the Nelson–Aalen estimator is consistent and converges weakly

$$\sqrt{n}\left(\hat{A}-A\right)\overset{D}{⟶}U={\left(U_{gh}\right)}_{g,h\in G}\;\;\text{on}\;\;D{\left[0,\tau \right]}^{\left(K\times K\right)},$$

where $D{\left[0,\tau \right]}^{\left(K\times K\right)}$ is the matrix‐valued càdlàg function space on [0,τ], that is, the space of $R^{\left(K\times K\right)}$‐valued càdlàg functions on [0,τ], equipped with the supremum norm and the σ‐field generated by supremum norm open balls. Here, the non‐diagonal entries of U are independent Gaussian martingales with $U_{gh}\left(0\right)=0$ and continuous sample paths. The diagonal entries of U are such that the row sums are zero. It also holds that

(3) $$\begin{array}{cc} & \sqrt{n}\left\{{\hat{A}}_{gh}\left(t\right)-A_{gh}\left(t\right)\right\}\\ & \;-\sqrt{n}\left[\int_{0}^{t}\frac{1\{Y_{g}\left(u\right)>0\}}{Y_{g}\left(u\right)}d\left\{\sum_{i=1}^{n}M_{i;gh}\left(u\right)\right\}\right]\overset{P}{\to }0, \end{array}$$

with $M_{i;gh}\left(t\right)=N_{i;gh}\left(t\right)-\int_{0}^{t}\alpha_{gh}\left(u\right)Y_{i;gh}\left(u\right)du$ a mean‐zero martingale.

This is used by the wild bootstrap in which the limiting distribution of $\sqrt{n}\left(\hat{A}-A\right)$ is approximated by replacing $dM_{i;gh}$ with $dN_{i;gh}\cdot G_{i;gh}$, with $G_{i;gh}$ a standard normal random variable. The random variables $G_{i;gh}$ are independent across i, g, and h. For this, the matrix ξ is calculated, where

$$\begin{array}{ccc} & & {\left(\xi \right)}_{gh}\left(t\right)=\xi_{gh}\left(t\right)\\ & & \;=\sqrt{n}\sum_{i=1}^{n}\int_{0}^{t}G_{i;gh}\left(u\right)\cdot 1\left\{Y_{g}\left(u\right)>0\right\}\frac{dN_{i;gh}\left(u\right)}{Y_{g}\left(u\right)},\;g\neq h, \end{array}$$

and diagonal elements such that the row sums are zero. Because of (3), ξ has the same limiting distribution as $\sqrt{n}\left(\hat{A}-A\right)$ (Bluhmki et al. 2018, Lin 1997).

Since the Kaplan–Meier estimator is a function of the Nelson–Aalen estimator, the wild bootstrap method for the Nelson–Aalen estimator can be transformed via a functional delta method argument for the Kaplan–Meier estimator and thus for a linear combination of Kaplan–Meier estimators. To approximate the limiting distribution of $\sqrt{n}\left\{\hat{\text{CGRFS}}\left(t\right)-\text{CGRFS}\left(t\right)\right\}$ with the wild bootstrap, it is first written as a function of the Nelson–Aalen estimator and the cumulative hazard matrix:

$$\begin{array}{cc} B(t) & =\sqrt{n}\left\{\hat{\text{CGRFS}}\left(t\right)-\text{CGRFS}\left(t\right)\right\}\\ & =\sqrt{n}[{\hat{S}}_{1}\left(t\right)+{\hat{S}}_{2}\left(t\right)-{\hat{S}}_{3}\left(t\right)+{\hat{S}}_{4}\left(t\right)-{\hat{S}}_{5}\left(t\right)-\{S_{1}\left(t\right)\\ & \;+S_{2}\left(t\right)-S_{3}\left(t\right)+S_{4}\left(t\right)-S_{5}\left(t\right)\}]\\ & =\sqrt{n}(S\left\{{\hat{f}}_{1}\left(\hat{A}\right)\right\}+S\left\{{\hat{f}}_{2}\left(\hat{A}\right)\right\}-S\left\{{\hat{f}}_{3}\left(\hat{A}\right)\right\}+S\left\{{\hat{f}}_{4}\left(\hat{A}\right)\right\}-S\left\{{\hat{f}}_{5}\left(\hat{A}\right)\right\}\\ & \;-[S\left\{f_{1}\left(A\right)\right\}+S\left\{f_{2}\left(A\right)\right\}-S\left\{f_{3}\left(A\right)\right\}+S\left\{f_{4}\left(A\right)\right\}-S\left\{f_{5}\left(A\right)\right\}]), \end{array}$$

which is asymptotically equal to

(4) $$\begin{array}{cc} & \sqrt{n}(S\left\{f_{1}\left(\hat{A}\right)\right\}+S\left\{f_{2}\left(\hat{A}\right)\right\}-S\left\{f_{3}\left(\hat{A}\right)\right\}+S\left\{f_{4}\left(\hat{A}\right)\right\}-S\left\{f_{5}\left(\hat{A}\right)\right\}\\ & \;-[S\left\{f_{1}\left(A\right)\right\}+S\left\{f_{2}\left(A\right)\right\}-S\left\{f_{3}\left(A\right)\right\}+S\left\{f_{4}\left(A\right)\right\}-S\left\{f_{5}\left(A\right)\right\}])\\ =: & \sqrt{n}\left\{\phi \left(\hat{A}\right)-\phi \left(A\right)\right\} \end{array}$$

with 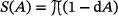 and $f_{j}\left(A\right)=u_{j}^{T}\cdot A\cdot v_{j}$ as in Section 3. With the functional delta method, it holds that

(5) $$\begin{array}{cc} B\;\overset{D}{\to }\;d\phi \left(A\right)\cdot U=\; & dS\left\{f_{1}\left(A\right)\right\}\cdot U+dS\left\{f_{2}\left(A\right)\right\}\cdot U\;-dS\left\{f_{3}\left(A\right)\right\}\\ & \cdot U+dS\left\{f_{4}\left(A\right)\right\}\cdot U-dS\left\{f_{5}\left(A\right)\right\}\cdot U, \end{array}$$

where dϕ(A)·U denotes the Hadamard derivative of ϕ(A). As ϕ(A) is a linear combination of five functions, its Hadamard derivative is the linear combination of the Hadamard derivatives $dS\{f_{j}\left(A\right)\}\cdot U$ of these five functions. For $dS\{f_{j}\left(A\right)\}\cdot U$ it holds that

(6) $$\left[dS\left\{f_{j}\left(A\right)\right\}\cdot U\right]\left(t\right)=-S_{j}\left(t\right)\int_{0}^{t}u_{j}^{T}\left(s\right)\cdot dU\left(s\right)\cdot v_{j}\left(s\right),$$

with $u_{j}\left(s\right)$ and $v_{j}\left(s\right)$ being the same as in $f_{j}\left(A\right)=u_{j}^{T}\cdot A\cdot v_{j}$. Plugging this result into Equation (5) and replacing dU with dξ, $y_{g}\left(t\right)$ with $Y_{g}\left(t\right)$, g=0,⋯,4, and $S_{j}$ with ${\hat{S}}_{j}$, j=1,⋯,5, yields an estimator $\hat{B}$ which approximates the limiting distribution of $\sqrt{n}\left\{\hat{\text{CGRFS}}\left(t\right)-\text{CGRFS}\left(t\right)\right\}$. The derivation of Equation (6) can be found in the Appendix.

## 6 Simultaneous Confidence Bands and Confidence Intervals

In this paper, simultaneous confidence bands and confidence intervals for chronic GvHD‐ and relapse‐free survival are generated using the wild bootstrap and Efron's bootstrap. Efron's bootstrap is performed by drawing units from the original dataset with replacement, with each unit having the same probability of being drawn. This bootstrap depends on an independently and identically distributed data structure (Davison and Hinkley 1997). This prerequisite is fulfilled for survival data if censoring is random, but general independent censoring does not suffice. The wild bootstrap, on the other hand, generates bootstrap realizations by replacing the unknown martingale increments with standard normal random variables as described in Section 5. It does not have the prerequisite of an i.i.d. data structure and is thus applicable in the case of independent censoring, as long as the Markov assumption is fulfilled. In the non‐Markov case, inference also depends on an i.i.d. data structure and therefore on random censoring (Nießl et al. 2023).

To obtain transformed simultaneous confidence bands for chronic GvHD‐ and relapse‐free survival estimated with a linear combination of Kaplan–Meier estimators, we adapted the wild bootstrap approach for the Aalen–Johansen estimator of Bluhmki et al. (2018). For this, a weighted and transformed variant of B is used, namely

$$C\left(t\right)=\sqrt{n}\cdot g\left(t\right)\left[h\left\{\hat{\text{CGRFS}}\left(t\right)\right\}-h\left\{\text{CGRFS}\left(t\right)\right\}\right],$$

with h(·) a transformation with nonzero continuous derivative dh(·) and g(·) a weight function. In the following sections, h(·) and g(·) are chosen as by Bluhmki et al. (2018), that is, h(x) as the log–log transformation log{−log(1−x)} and the weight function as

$$g\left(t\right)=\frac{\left\{\hat{\text{CGRFS}}\left(t\right)-1\right\}\cdot \log\left\{1-\hat{\text{CGRFS}}\left(t\right)\right\}}{\sqrt{n\cdot \hat{\text{var}}\left\{\hat{\text{CGRFS}}\left(t\right)\right\}}},$$

where $\hat{\text{var}}\left\{\hat{\text{CGRFS}}\left(t\right)\right\}$ is the empirical variance of the bootstrap realizations divided by *n* in the case of the wild bootstrap and the empirical variance of the bootstrap realizations in the case of Efron's bootstrap. With the functional delta method, it holds that

$$\hat{C}\left(t\right)=g\left(t\right)\cdot dh\left\{\hat{\text{CGRFS}}\left(t\right)\right\}\cdot \hat{B}\left(t\right)$$

approximates the limiting distribution of C. Here, $\hat{B}\left(t\right)$ is the wild bootstrap realization which approximates the limiting distribution of the linear combination of Kaplan–Meier estimators. When using Efron's bootstrap, $\hat{B}\left(t\right)$ corresponds to $\sqrt{n}\left\{{\hat{\text{CGRFS}}}^{*}\left(t\right)-\hat{\text{CGRFS}}\left(t\right)\right\}$, with ${\hat{\text{CGRFS}}}^{*}\left(t\right)$ the bootstrap realization of chronic GvHD‐ and relapse‐free survival.

Let $q_{\alpha }^{CB}$ denote the (1 −α) quantile of ${\text{sup}}_{t\in [t_{1},t_{2}]}\left|\hat{C}\left(t\right)\right|$, α∈(0,1). Then the asymptotic (1 − α) confidence band of h{CGRFS(t)} in the time interval $\left[t_{1},t_{2}\right]$ is given by

$$h\left\{\hat{\text{CGRFS}}\left(t\right)\right\}\pm \frac{q_{\alpha }^{CB}}{\sqrt{n}\cdot g\left(t\right)},\;t\in \left[t_{1},t_{2}\right].$$

By retransforming through applying the inverse function $h^{-1}\left(\cdot \right)$, one obtains an asymptotic (1−α) confidence band for chronic GvHD‐ and relapse‐free survival.

Confidence bands can also be obtained for two‐group comparisons. Bluhmki et al. (2018) describe an approach to obtain confidence bands for the differences of two independent samples with the wild bootstrap that can be easily applied to chronic GvHD‐ and relapse‐free survival estimated with a linear combination of Kaplan–Meier estimators.

A (1−α) log–log confidence interval for chronic GvHD‐ and relapse‐free survival at time point t is given by

$$\begin{array}{cc} & 1-{\left\{1-\hat{\text{CGRFS}}\left(t\right)\right\}}^{\theta },\;\theta \\ & \;=\exp\left[\pm \frac{q_{\alpha /2}^{CI}}{\left\{\hat{\text{CGRFS}}\left(t\right)-1\right\}\cdot \log\left\{1-\hat{\text{CGRFS}}\left(t\right)\right\}}\right]. \end{array}$$

Like $q_{\alpha }^{CB}$, $q_{\alpha /2}^{CI}$ depends on the bootstrap method used to obtain the confidence interval. In the case of the wild bootstrap, $q_{\alpha /2}^{CI}$ is the (1−α/2) quantile of $\hat{B}\left(t\right)/\sqrt{n}$. In the case of Efron's bootstrap, $q_{\alpha /2}^{CI}$ is the (1−α/2) quantile of ${\hat{\text{CGRFS}}}^{*}\left(t\right)-\hat{\text{CGRFS}}\left(t\right)$.

## 7 Chronic GvHD‐ and Relapse‐Free Survival in the German Registry for Hematopoietic Stem Cell Transplantation and Cell Therapy

In this section, differences between the confidence bands of the three estimators are examined using data from 628 patients, provided by the German Registry for Hematopoietic Stem Cell Transplantation and Cell Therapy (DRST). To test the Markov assumption in the illness–death model with recovery, the number of previous episodes of chronic GvHD was added as a covariate in an Andersen–Gill model. In the progressive six‐state model, the entry time into state g was added as a covariate in a Prentice–Williams–Peterson model for the test of the transitions out of state g, ∀g∈G. Both tests showed that the Markov assumption should be rejected with *p*‐values smaller than 0.001.

**FIGURE 2:**
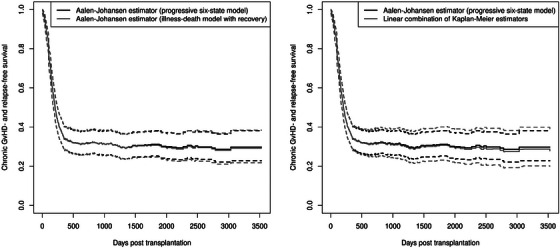
Pairwise comparison between the Aalen–Johansen estimator in the progressive six‐state model (black line in both panels) and the Aalen–Johansen estimator in the illness–death model with recovery (gray line in the left panel) and the linear combination of Kaplan–Meier estimators (gray line in the right panel). The log–log transformed 95% confidence bands are represented by dashed lines.

The data were used to estimate chronic GvHD‐ and relapse‐free survival with the three estimators. Log–log transformed confidence bands were obtained using the wild bootstrap with 1000 iterations for each estimator. Figure 2 shows the pairwise comparison between the Aalen–Johansen estimator in the progressive six‐state model and the two other estimators together with their respective confidence bands. The Aalen–Johansen estimator in the illness–death model with recovery and the Aalen–Johansen estimator in the progressive six‐state model give similar results, as can be seen on the left‐hand side of Figure 2. Small differences in the confidence bands are visible starting at 2000 days after transplantation.

The comparison of the linear combination of Kaplan–Meier estimators and the Aalen–Johansen estimator in the progressive six‐state model on the right‐hand side of Figure 2 shows slightly more of a difference between the confidence bands of the two estimators. The deviations also occur at an earlier point in time, from about 500 days after transplantation. The confidence band of the linear combination of Kaplan–Meier estimators is wider than that of the Aalen–Johansen estimator in the progressive six‐state model and, due to the similar results of the two Aalen–Johansen estimators, also wider than the confidence band of the Aalen–Johansen estimator in the illness death model with recovery.

## 8 Simulations

In order to further examine the differences between the linear combination of Kaplan–Meier estimators and the two Aalen–Johansen estimators, the coverage probabilities of confidence intervals and confidence bands generated by Efron's bootstrap and by the wild bootstrap in a non‐Markov scenario were analyzed. To compute the coverage probabilities, simulations were used (Beyersmann et al. 2012, Bluhmki, Putter et al. 2019). The simulations were conducted with sample sizes *n* = 200, *n* = 400, and *n* = 1000 and are based on the progressive six‐state model, in which the hazards are assumed to be constant given an individual frailty. The hazards were defined as $\alpha_{01}=0.0009$, $\alpha_{12}=0.0008$, $\alpha_{23}=0.001$, $\alpha_{34}=0.00065$, $\alpha_{05}=0.00075$, and $\alpha_{15}=\alpha_{25}=\alpha_{35}=\alpha_{45}=0.0006$. The random censoring time C was chosen to be exponentially distributed with parameter 0.00095.

To ensure that the Markov assumption does not hold in the simulations, the hazards were multiplied with a gamma frailty with mean and variance equal to two, that is, $\alpha_{i,gh}=Z_{i}\alpha_{gh},\;Z_{i}∼G\left(2,1\right),\;g\in \left\{0,\cdots ,4\right\},\;h\in \left\{1,\cdots ,5\right\},\;g\neq h,\;i=1,\cdots ,n$. In the illness–death model with recovery, a test detected a violation of the Markov assumption in 77.8% of the datasets with 200 patients. In the progressive six‐state model, a violation was detected in 14.4% of the simulated datasets with 200 patients. The fact that the Markov assumption in the illness–death model is more often rejected by tests than in the progressive model is not too surprising. In the progressive six‐state model, the Markov assumption is only violated by the gamma frailty; that is, the Markov assumption is fulfilled as soon as the frailty term is omitted. However, for the Markov assumption to be fulfilled in the illness–death model with recovery, additionally, all hazards from a state “Alive and healthy” to a state “Alive and ill” would have to have the same value in the simulation, that is, $\alpha_{01}=\alpha_{23}$ has to hold, which is not the case. Likewise, the Markov assumption in the illness–death model with recovery is violated because $\alpha_{12}\neq \alpha_{34}$ and $\alpha_{05}\neq \alpha_{25}=\alpha_{45}$.

For each sample size, 1000 simulations were performed. The true probabilities were approximated using the Aalen–Johansen estimator in the progressive six‐state model in a simulated dataset without right‐censoring with 200,000 individuals. For each simulated dataset, the wild bootstrap and Efron's bootstrap were performed with 1000 iterations each. The resulting bootstrap realizations were used to construct a confidence interval and a confidence band for each dataset. The coverage probability was calculated as the proportion of the confidence intervals, respectively, confidence bands which contained the true values. One issue that has arisen in the construction of confidence intervals and confidence bands in the simulated datasets is that the linear combination of Kaplan–Meier estimators may estimate probabilities to be negative or greater than one. As a workaround, all negative values have been transformed to zero and all values greater than one to one, so that the chronic GvHD‐ and relapse‐free survival estimated with the linear combination of Kaplan–Meier estimators on the simulated datasets and the bootstrapped datasets obtained with Efron's bootstrap stays within the interval [0,1]. The bootstrap realizations obtained with the wild bootstrap are not affected by this transformation, but the transformed chronic GvHD‐ and relapse‐free survival plays a role in the construction of the confidence bands and intervals. As a consequence, the confidence intervals and confidence bands for the linear combination of Kaplan–Meier estimators obtained with both bootstrap methods slightly differ from the intervals and bands that would have been obtained if the transformation had not been applied.

Table 3 shows the coverage probabilities of the confidence intervals for each sample size and for each of the three estimators at the time points 500, 750, 1000, and 2000. Regarding the coverage probabilities of the confidence intervals obtained with Efron's bootstrap, all three estimators yield comparable results. Their values range from 89.4% to 95.8% for the Aalen–Johansen estimator in the illness–death model with recovery, from 89.8% to 95.8% for the Aalen–Johansen estimator in the progressive six‐state model, and from 90.2% to 96.1% for the linear combination of Kaplan–Meier estimators. Here, the coverage probabilities at the time points 500, 750, and 1000 ranged around 95%, but for the time point 2000 they tend to be lower. This is because only few patients are left in the dataset at this time point.

**TABLE 3:** Coverage probabilities of log–log transformed 95% confidence intervals in simulations based on the progressive‐six state model.

		Efron's bootstrap				Wild bootstrap			
*n*	Estimator	*t* = 500	*t* = 750	*t* = 1000	*t* = 2000	*t* = 500	*t* = 750	*t* = 1000	*t* = 2000
200	AJE (ill‐death)	0.955	0.948	0.955	0.895	0.953	0.951	0.946	0.893
	AJE (prog)	0.956	0.945	0.947	0.898	0.951	0.945	0.937	0.852
	KME comb	0.958	0.949	0.947	0.902	0.983	0.981	0.985	0.967
400	AJE (ill‐death)	0.94	0.945	0.954	0.943	0.94	0.949	0.952	0.927
	AJE (prog)	0.939	0.941	0.953	0.926	0.937	0.938	0.948	0.882
	KME comb	0.935	0.942	0.954	0.945	0.97	0.984	0.985	0.977
1000	AJE (ill‐death)	0.954	0.958	0.958	0.936	0.958	0.956	0.965	0.932
	AJE (prog)	0.953	0.958	0.958	0.935	0.958	0.956	0.956	0.911
	KME comb	0.956	0.961	0.961	0.931	0.98	0.991	0.988	0.98

*Note:* The confidence intervals were generated with Efron's bootstrap and the wild bootstrap with 1000 iterations. The coverage probabilities were calculated separately for each sample size and the time points t, as well as the Aalen–Johansen estimator in the illness–death model with recovery (AJE (ill‐death)), the Aalen–Johansen estimator in the progressive six‐state model (AJE (prog)) and the linear combination of Kaplan–Meier estimators with negative values transformed to zero (KME comb).

Regarding the coverage probabilities of the confidence intervals which were obtained with the wild bootstrap, the two Aalen–Johansen estimators give comparable results, while the linear combination of Kaplan–Meier estimators results in greater coverage probabilities. The coverage probabilities for the linear combinations of Kaplan–Meier estimators range from 96.7% to 99.1%, while the coverage probabilities of the Aalen–Johansen estimators range from 85.2% to 95.8%. This suggests that the wild bootstrap approach for the linear combination of Kaplan–Meier estimators produces wider confidence intervals than the wild bootstrap for the two Aalen–Johansen estimators. This is in line with the results in Section 7, where the confidence bands of the linear combination of Kaplan–Meier estimators obtained with the wild bootstrap were wider than the confidence bands of the two Aalen–Johansen estimators.

To compare our wild bootstrap approach with the wild bootstrap approach of Liu et al. (2008), we simulated data for the progressive four‐state model described by Liu et al. (2008) and adapted our approach to this smaller model. We then generated bootstrap realizations with both approaches and used them to calculate confidence intervals and their coverage probabilities. The coverage probabilities of the confidence intervals generated with our approach were comparable to those obtained with the approach of Liu et al. (2008).

Additionally, we analyzed the coverage probabilities of log–log transformed 95% confidence bands over the time intervals [$t_{1}$,500], [$t_{1}$,750], [$t_{1}$,1000], and [$t_{1}$,2000] with starting points $t_{1}=0$ and $t_{1}=10$ for each sample size. Also, the coverage probabilities of log–log transformed 95% confidence bands over the time intervals with starting points $t_{1}=50$, $t_{1}=100$, and $t_{1}=250$ were obtained in the datasets with 200 individuals.

The coverage probabilities of the confidence bands over the time intervals with starting point $t_{1}=0$ are shown in Table 4. The coverage probabilities are smaller than 95% for all three estimators and both bootstrap methods. They range from 85.7% to 93% for the Aalen–Johansen estimator in the illness–death model with recovery, from 85.3% to 93.2% for the Aalen–Johansen estimator in the progressive six‐state model, and from 90.6% to 93.7% for the linear combination of Kaplan–Meier estimators.

**TABLE 4:** Coverage probabilities of log–log transformed 95% confidence bands in simulations based on the progressive‐six state model.

		Efron's bootstrap				Wild bootstrap			
*n*	Estimator	[0,500]	[0,750]	[0,1000]	[0,2000]	[0,500]	[0,750]	[0,1000]	[0,2000]
200	AJE (ill‐death)	0.927	0.927	0.918	0.883	0.925	0.925	0.912	0.857
	AJE (prog)	0.927	0.926	0.92	0.903	0.923	0.917	0.907	0.853
	KME comb	0.929	0.927	0.921	0.906	0.936	0.937	0.934	0.93
400	AJE (ill‐death)	0.908	0.913	0.91	0.915	0.896	0.905	0.902	0.894
	AJE (prog)	0.91	0.912	0.906	0.913	0.896	0.893	0.891	0.867
	KME comb	0.912	0.914	0.913	0.918	0.92	0.927	0.926	0.929
1000	AJE (ill‐death)	0.925	0.93	0.916	0.913	0.899	0.907	0.904	0.901
	AJE (prog)	0.923	0.932	0.912	0.917	0.903	0.909	0.908	0.899
	KME comb	0.925	0.933	0.916	0.918	0.913	0.924	0.928	0.932

*Note:* The confidence bands were generated with Efron's bootstrap and the wild bootstrap with 1000 iterations. The coverage probabilities were calculated separately for each sample size and the time intervals [0, t], as well as the Aalen–Johansen estimator in the illness–death model with recovery (AJE (ill‐death)), the Aalen–Johansen estimator in the progressive six‐state model (AJE (prog)) and the linear combination of Kaplan–Meier estimators with negative values transformed to zero (KME comb).

Table 5 shows the coverage probabilities of confidence bands over the time intervals with starting point $t_{1}$ = 10. In the datasets with 200 and 1000 patients, the coverage probabilities of the confidence bands of the Aalen–Johansen estimators are similar for both bootstrap methods and range around 95%. The coverage probabilities of the confidence bands of the linear combination of Kaplan–Meier estimators obtained with Efron's bootstrap are comparable, but the coverage probabilities obtained with the wild bootstrap are greater than 95%. The coverage probabilities in the dataset with 400 patients are lower than those in the datasets with 200 or 1000 patients for all three estimators and both bootstrap methods. These results are consistent with the coverage probabilities of the confidence intervals and indicate that the confidence bands of the linear combination of Kaplan–Meier estimators are wider than those of the two Aalen–Johansen estimators, as seen in Figure 2.

**TABLE 5:** Coverage probabilities of log–log transformed 95% confidence bands in simulations based on the progressive‐six state model.

		Efron's bootstrap				Wild bootstrap			
*n*	Estimator	[10,500]	[10,750]	[10,1000]	[10,2000]	[10,500]	[10,750]	[10,1000]	[10,2000]
200	AJE (ill‐death)	0.954	0.95	0.939	0.897	0.951	0.948	0.941	0.881
	AJE (prog)	0.953	0.948	0.937	0.92	0.946	0.941	0.935	0.868
	KME comb	0.953	0.948	0.94	0.919	0.967	0.965	0.963	0.957
400	AJE (ill‐death)	0.929	0.928	0.923	0.917	0.923	0.927	0.923	0.914
	AJE (prog)	0.926	0.928	0.926	0.913	0.918	0.922	0.914	0.885
	KME comb	0.926	0.93	0.93	0.922	0.953	0.957	0.957	0.957
1000	AJE (ill‐death)	0.952	0.953	0.948	0.942	0.947	0.947	0.944	0.939
	AJE (prog)	0.951	0.954	0.948	0.942	0.951	0.948	0.942	0.936
	KME comb	0.95	0.956	0.952	0.943	0.965	0.969	0.976	0.979

*Note:* The confidence bands were generated with Efron's bootstrap and the wild bootstrap with 1000 iterations. The coverage probabilities were calculated separately for each sample size and the time intervals [10, t], as well as the Aalen–Johansen estimator in the illness–death model with recovery (AJE (ill‐death)), the Aalen–Johansen estimator in the progressive six‐state model (AJE (prog)) and the linear combination of Kaplan–Meier estimators with negative values transformed to zero (KME comb).

The coverage probabilities of confidence bands over the time intervals with start points t= 50, t= 100, and t= 250 in the simulated datasets with 200 patients are shown in Table 6. Despite the later start points of the time intervals, the coverage probabilities also range around 95%, except for the confidence bands of the linear combination of Kaplan–Meier estimators obtained with the wild bootstrap.

**TABLE 6:** Coverage probabilities of log–log transformed 95% confidence bands in simulations based on the progressive‐six state model with *n* = 200.

		Efron's bootstrap				Wild bootstrap			
$t_{1}$	Estimator	[$t_{1}$,500]	[$t_{1}$,750]	[$t_{1}$,1000]	[$t_{1}$,2000]	[$t_{1}$,500]	[$t_{1}$,750]	[$t_{1}$,1000]	[$t_{1}$,2000]
50	AJE (ill‐death)	0.955	0.954	0.947	0.898	0.953	0.952	0.944	0.881
	AJE (prog)	0.952	0.95	0.946	0.92	0.951	0.944	0.934	0.867
	KME comb	0.954	0.952	0.945	0.922	0.974	0.972	0.969	0.962
100	AJE (ill‐death)	0.958	0.957	0.949	0.903	0.958	0.954	0.947	0.883
	AJE (prog)	0.958	0.956	0.948	0.922	0.957	0.948	0.937	0.867
	KME comb	0.961	0.957	0.947	0.927	0.979	0.979	0.976	0.966
250	AJE (ill‐death)	0.946	0.944	0.944	0.898	0.948	0.94	0.937	0.874
	AJE (prog)	0.945	0.947	0.944	0.914	0.941	0.932	0.928	0.851
	KME comb	0.948	0.946	0.946	0.925	0.98	0.983	0.981	0.97

*Note:* The confidence bands were generated with Efron's bootstrap and the wild bootstrap with 1000 iterations. The coverage probabilities were calculated separately for the time intervals [$t_{1},t$], as well as the Aalen–Johansen estimator in the illness–death model with recovery (AJE (ill‐death)), the Aalen–Johansen estimator in the progressive six‐state model (AJE (prog)) and the linear combination of Kaplan–Meier estimators with negative values transformed to zero (KME comb).

The fact that the coverage probabilities of the confidence bands over the time intervals with starting point $t_{1}=0$ in Table 4 are lower than 95% for both bootstrap methods and the fact that the coverage probabilities of the confidence bands over time intervals with later starting points range around 95% lead to the conclusion that the reason for the low coverage probabilities is not the violation of the Markov assumption but uncertainties in the estimation at the first few time points. These uncertainties are caused by too few transitions at the first time points after transplantation.

## 9 Discussion

Suitable estimators for probabilities of complex multistate outcomes with recurrent events in non‐Markov models are required in many applications, for example, pseudo‐value regression (Andersen et al. 2003) or estimation of marginal features as the mean number of recurrent events (Cortese and Scheike 2022; Erdmann et al. 2023). One concern when the Markov assumption is not fulfilled was the consistency of the Aalen–Johansen estimator, but it was proven that it is consistent even in non‐Markov scenarios, as long as state occupation probabilities are estimated and censoring is random (Datta and Satten 2001; Overgaard 2019; Nießl et al. 2023).

Nevertheless, which estimator is best suited to estimate complex outcomes in non‐Markov settings remains an important research question. Therefore, we compare the Aalen–Johansen estimator in the illness–death model with recovery with a linear combination of Kaplan–Meier estimators and the Aalen–Johansen estimator in the progressive six‐state model in a non‐Markov setting. Our comparison can provide new insights, since in the past only few simulations of non‐Markov scenarios have been conducted, and in those mostly the illness–death model without recovery was considered.

In a real dataset from the German Registry for Hematopoietic Stem Cell Transplantation and Cell Therapy, the comparison of the estimated chronic GvHD‐ and relapse‐free survival showed only very small differences between the Aalen–Johansen estimators in the two different models and the linear combination of the Kaplan–Meier estimators, despite tests indicating the Markov assumption to not be fulfilled in both models. This shows that a violation of the Markov assumption does not seem to affect the Aalen–Johansen estimator in either model and affirms its consistency for state occupation probabilities if censoring is random.

We also compared confidence bands and confidence intervals for the three estimators, which were obtained using Efron's bootstrap and the wild bootstrap. The wild bootstrap is a practical bootstrap method when dealing with survival data, as it resamples martingale errors. Furthermore, it provides reliable results not only if censoring is random but also with more general censoring schemes in settings where the Markov assumption is fulfilled (Nießl et al. 2023; Rühl et al. 2023).

Proposals for wild bootstrap approaches for linear combinations of Kaplan–Meier estimators already exist in the literature (Liu et al. 2008; Zhang et al. 2022). But these approaches have been constructed for specific linear combinations and multistate models. In contrast, our method provides a general approach which can be adapted easily to different multistate models and their respective linear combinations of Kaplan–Meier estimators. There are differences between the wild bootstrap approaches of Liu et al. (2008) and Zhang et al. (2022) with respect to the drawing of the standard normal random variables. In the approach of Liu et al. (2008) and our approach, a random variable is drawn for every observed transition at the observed event times. Zhang et al. (2022), on the other hand, draw one random variable per observed event and per Kaplan–Meier estimator for which the event is relevant. Different approaches when drawing the random variables were discussed by Bluhmki, Dobler, et al. (2019), with a preference for drawing one random variable per transition if resampling is based on a martingale representation.

The coverage probabilities of the confidence intervals and confidence bands obtained with Efron's bootstrap and the wild bootstrap were investigated by simulating a non‐Markov scenario. The coverage probabilities of the confidence intervals obtained with Efron's bootstrap were comparable for all three estimators and ranged around 95%. The coverage probabilities of the confidence intervals obtained with the wild bootstrap were also similar for the two Aalen–Johansen estimators and close to the coverage probabilities obtained with Efron's bootstrap. The coverage probabilities of the confidence intervals for the linear combination of Kaplan–Meier estimators, on the other hand, were noticeably greater than those of the Aalen–Johansen estimator and greater than 95% when using the wild bootstrap. Similar results are observed for the coverage probabilities of the confidence bands in simulated datasets. All these results, together with the analysis of the real data example, show that the confidence intervals and confidence bands obtained with the wild bootstrap for the linear combination of the Kaplan–Meier estimators are wider than those of the two Aalen–Johansen estimators.

The wide confidence intervals and confidence bands of the linear combination of Kaplan–Meier estimators obtained with the wild bootstrap probably arise because this estimator sometimes estimates values to be greater than one or negative. For the Aalen–Johansen estimators, the coverage probabilities of the confidence intervals and bands obtained with the wild bootstrap are close to the coverage probabilities of those obtained with Efron's bootstrap, which indicates that the wild bootstrap is not affected by a violation of the Markov assumption if censoring is random. Nießl et al. (2023) found similar results for (landmark) Aalen–Johansen estimation with comparable coverages for both Efron's and wild bootstrap in a randomly left‐truncated illness–death model without recovery, with a slight preference for the wild bootstrap. If, however, a Markov assumption is justified, the wild bootstrap allows for more general censoring schemes, and, for example, Nießl et al. (2023) and Rühl et al. (2023) demonstrate that drawing with replacement from the units under study may lead to worse coverages when an i.i.d. structure is violated by event‐driven censoring.

Altogether, our results show that a violation of the Markov assumption does not seem to affect the Aalen–Johansen estimator nor the wild bootstrap if censoring is random. This indicates that the Aalen–Johansen estimator is suitable for estimating probabilities of complex outcomes even in the non‐Markov case, especially since the linear combination of Kaplan–Meier estimators can estimate probabilities to be negative or greater than one. Consequently, the illness–death model with recovery is again of interest for estimating complex probabilities with recurrent events. This is especially appealing for studies where only limited data are available, since this model, due to the division of the individuals into only two states and subsequent estimation of fewer quantities, uses more data for the estimation of probabilities than progressive models. However, it must be noted that in the non‐Markov case the Aalen–Johansen estimator only consistently estimates state occupation probabilities and that only if censoring is random. If censoring is not random or transition probabilities are of interest, alternative estimators must be used in non‐Markov settings. One alternative for estimating transition probabilities in non‐Markov settings with random censoring is the landmark Aalen–Johansen estimator (Putter and Spitoni 2018), which was extended by Maltzahn et al. (2021) to the hybrid landmark Aalen–Johansen estimator. Andersen et al. (2022) compare several estimators for transition probabilities in a non‐Markov illness‐death model without recovery. The Aalen–Johansen estimator was biased as expected, but the landmark Aalen–Johansen estimator was consistent and comparable to a landmark Pepe estimator.

## Ethics Approval Obtained

Only anonymized clinical cohort data are used. An approval from the ethical board of Ulm University was obtained (226/17, 141/19).

## Conflicts of Interest

The authors declare no conflicts of interest.

## Supporting Information

Additional Supporting Information including source code to reproduce the results may be found online in the supporting information tab for this article. Some of the functions are based on the supplementary code of Bluhmki et al. (2018).

## Open Research Badges

This article has earned an Open Data badge for making publicly available the digitally‐shareable data necessary to reproduce the reported results. The data is available in the Supporting Information section.

This article has earned an open data badge “**Reproducible Research**” for making publicly available the code necessary to reproduce the reported results. The results reported in this article were reproduced partially due to data confidentiality issues.

## Supporting information


**Supporting File:** bimj70082o‐supo‐0001o‐Code.zip.

## Data Availability

The data underlying this article were provided by the German Registry for Hematopoietic Stem Cell Transplantation and Cell Therapy (Deutsches Register für hämatopoetische Stammzelltransplantation und Zelltherapie, DRST). Data will be shared on request to the corresponding author with permission of DRST and in accordance with their data sharing procedures.

## Appendix A

### A.1 Proof of

We will show the convergence in probability by using ${\hat{S}}_{3}\left(t\right)$ as an example, but the same arguments hold for the other four Kaplan–Meier estimators. To show that

it suffices to show that

(A1)
$$\sqrt{n}\int_{0}^{t}\left\{\frac{Y_{0}\left(u\right)}{Y_{0}\left(u\right)+Y_{1}\left(u\right)}-\frac{y_{0}\left(u\right)}{y_{0}\left(u\right)+y_{1}\left(u\right)}\right\}d{\hat{A}}_{05}\left(u\right)\overset{P}{\to }0$$

and

(A2)
$$\sqrt{n}\int_{0}^{t}\left\{\frac{Y_{1}\left(u\right)}{Y_{0}\left(u\right)+Y_{1}\left(u\right)}-\frac{y_{1}\left(u\right)}{y_{0}\left(u\right)+y_{1}\left(u\right)}\right\}d\left({\hat{A}}_{15}+{\hat{A}}_{12}\right)\left(u\right)\overset{P}{\to }0.$$

In the following, we will show the convergence in probability of Equation (A1). The convergence of (A2) can be proven analogously.

Let η>0. It holds that

(A3)
$$\begin{array}{cc} & P\left[\int_{0}^{t}\sqrt{n}\left\{\frac{Y_{0}\left(u\right)}{Y_{0}\left(u\right)+Y_{1}\left(u\right)}-\frac{y_{0}\left(u\right)}{y_{0}\left(u\right)+y_{1}\left(u\right)}\right\}d{\hat{A}}_{05}\left(u\right)>\eta \right]\\ & =P\left[\int_{0}^{t}\sqrt{n}\left\{\frac{Y_{0}\left(u\right)}{Y_{0}\left(u\right)+Y_{1}\left(u\right)}-\frac{y_{0}\left(u\right)}{y_{0}\left(u\right)+y_{1}\left(u\right)}\right\}\frac{dN_{05}\left(u\right)}{Y_{0}\left(u\right)}>\eta \right]\\ & \leq P\left[\underset{t\in [0,\tau ]}{\sup}\left\{\int_{0}^{t}\sqrt{n}\left|\frac{Y_{0}\left(u\right)}{Y_{0}\left(u\right)+Y_{1}\left(u\right)}-\frac{y_{0}\left(u\right)}{y_{0}\left(u\right)+y_{1}\left(u\right)}\right|\frac{dN_{05}\left(u\right)}{Y_{0}\left(u\right)}\right\}>\eta \right]. \end{array}$$

Since $\sqrt{n}\left|\frac{Y_{0}\left(u\right)}{Y_{0}\left(u\right)+Y_{1}\left(u\right)}-\frac{y_{0}\left(u\right)}{y_{0}\left(u\right)+y_{1}\left(u\right)}\right|\frac{1}{Y_{0}\left(u\right)}$ is nonnegative and predictable, one can use Lenglart's inequality to obtain that (A3) is smaller than or equal to

(A4)
$$P\left\{\int_{0}^{t}\sqrt{n}\left|\frac{Y_{0}\left(u\right)}{Y_{0}\left(u\right)+Y_{1}\left(u\right)}-\frac{y_{0}\left(u\right)}{y_{0}\left(u\right)+y_{1}\left(u\right)}\right|\frac{d\Lambda_{05}\left(u\right)}{Y_{0}\left(u\right)}>\delta \right\}+\frac{\delta }{\eta },$$

where δ>0 and $\Lambda_{05}\left(t\right)=\int_{0}^{t}\lambda_{05}\left(u\right)du$ is the compensator of $N_{05}\left(t\right)$ with $\lambda_{05}\left(t\right)=Y_{0}\left(t\right)\alpha_{05}\left(t\right)$ is the intensity process of this counting process. For (A4), it holds that

(A5)
$$\begin{array}{cc} & \int_{0}^{t}\sqrt{n}\left|\frac{Y_{0}\left(u\right)}{Y_{0}\left(u\right)+Y_{1}\left(u\right)}-\frac{y_{0}\left(u\right)}{y_{0}\left(u\right)+y_{1}\left(u\right)}\right|\frac{d\Lambda_{05}\left(u\right)}{Y_{0}\left(u\right)}\\ & =\int_{0}^{t}\sqrt{n}\left|\frac{Y_{0}\left(u\right)}{Y_{0}\left(u\right)+Y_{1}\left(u\right)}-\frac{y_{0}\left(u\right)}{y_{0}\left(u\right)+y_{1}\left(u\right)}\right|\frac{\lambda_{05}\left(u\right)}{Y_{0}\left(u\right)}du\\ & =\int_{0}^{t}\sqrt{n}\left|\frac{Y_{0}\left(u\right)}{Y_{0}\left(u\right)+Y_{1}\left(u\right)}-\frac{y_{0}\left(u\right)}{y_{0}\left(u\right)+y_{1}\left(u\right)}\right|\frac{Y_{0}\left(u\right)\alpha_{05}\left(u\right)}{Y_{0}\left(u\right)}du\\ & =\int_{0}^{t}\left|\sqrt{n}\frac{Y_{0}\left(u\right)}{Y_{0}\left(u\right)+Y_{1}\left(u\right)}-\sqrt{n}\frac{y_{0}\left(u\right)}{y_{0}\left(u\right)+y_{1}\left(u\right)}\right|\alpha_{05}\left(u\right)du\\ & \leq \int_{0}^{t}\left|n\frac{Y_{0}\left(u\right)}{Y_{0}\left(u\right)+Y_{1}\left(u\right)}-\frac{y_{0}\left(u\right)}{y_{0}\left(u\right)+y_{1}\left(u\right)}\right|\alpha_{05}\left(u\right)du, \end{array}$$

with the last inequality holding for n large enough. Now, it holds that (A5) converges to zero in probability, as

$$\begin{array}{c} \left|n\frac{Y_{0}\left(u\right)}{Y_{0}\left(u\right)+Y_{1}\left(u\right)}-\frac{y_{0}\left(u\right)}{y_{0}\left(u\right)+y_{1}\left(u\right)}\right|\overset{P}{\to }0, \end{array}$$

if ${\text{sup}}_{u\in [s,t]}\left|n^{-1}Y_{h}\left(u\right)-y_{h}\left(u\right)\right|\overset{P}{\to }0$, ${\text{inf}}_{u\in [s,t]}y_{h}\left(u\right)>0$, $\sqrt{n}\to \infty$, h=0,1, which is a usual regularity assumption.

### A.2 Hadamard Derivative of $S\{f_{j}\left(A\right)\}$

In this section, we will derive the Hadamard derivative of

$$S\{f_{j}\left(A\right)\},\;j=1,\cdots ,5,$$

with  and $f_{j}\left(A\right)=u_{j}^{T}\cdot A\cdot v_{j},\;j=1,\text{…},5$, with $u_{j}$ and $v_{j}$ chosen as in Section 3. It holds that the derivative of S(A) is equal to

Therefore, $[dS\left\{f_{j}\left(A\right)\right\}\cdot U]\left(t\right)$ in Equation (4) is equal to

(A6)

For the linear transformations $f_{j}\left(A\right)$, it holds that their Hadamard derivative is equal to

(A7)
$$\left\{df_{j}\left(A\right)\cdot U\right\}\left(t\right)=u_{j}^{T}\left(t\right)\cdot U\left(t\right)\cdot v_{j}\left(t\right),\;j=1,\cdots ,5.$$

Plugging Equation (A7) into Equation (A6) results in

$$\left[dS\left\{f_{j}\left(A\right)\right\}\cdot U\right]\left(t\right)=-S_{j}\left(t\right)\int_{0}^{t}u_{j}^{T}\left(s\right)\cdot dU\left(s\right)\cdot v_{j}\left(s\right),\;j=1,\cdots ,5.$$
