# Supplementary material for: Non‐Markov Nonparametric Estimation of Complex Multistate Outcomes After Hematopoietic Stem Cell Transplantation
Source: Biom J. 2025 Oct 29;67(6):e70082. doi: 10.1002/bimj.70082 (PMC12569748; doi:10.1002/bimj.70082)
Supplement: Supplementary file 1 — Supporting File: bimj70082o‐supo‐0001o‐Code.zip. [file BIMJ-67-e70082-s001.zip › Code_supplement/readme.pdf]

README

Code supplement for the manuscript "Non-Markov non-parametric estimation of complex multistate outcomes after hematopoietic stem cell transplantation" by J. Vilsmeier, S. Schmeller, D. Fürst and J. Beyersmann

For questions, comments or remarks about the code please contact J. Vilsmeier (judith.vilsmeier@uni-ulm.de).

The code has been written using R version 4.1.2 (platform x86_64-pc-linux-gnu) with packages parallel, data.table_1.14.4, survival_3.4-0 and etm_1.1.1.
A copy of the output of R’s sessionInfo() can be found on page 3.

The subfolder “Pseudo_data” contains a simulated dataset that mimics the main features of the real data analysed in the article.
To generate an example for Figure 2 run the first part of runme.R which uses pseudo data. The figure will be stored in the subfolder “Results”. All intermediate results will be stored in the subfolder “Pseudo_data”. The computation of the confidence bands uses B=1000 bootstrap iterations and it takes about 2 hours to run.

To reproduce the simulation results presented in the manuscript run the second part of runme.R. All tables will be stored in the subfolder “Results”. All intermediate results will be stored in the subfolders “Simulation_n=200”, “Simulation_n=400” and “Simulation_n=1000”.
The coverage probabilities shown in the manuscript were obtained using K = 1000 simulated datasets and B = 1000 bootstrap iterations. This takes several days to weeks to run. Reducing K and B will reduce the running time, however, the results will differ somewhat from the original tables.

The functions used for the simulations and computation of confidence intervals, confidence bands and coverage probabilities are provided in the subfolder “Functions” and subsequent subfolders (see folder structure below).
Some of the functions are based on functions provided in the supplementary code of Bluhmki et al. (2018) (https://academic.oup.com/biometrics/article/74/3/977/7526051#supplementary-data). These functions are provided in the subfolder “./Functions/Functions_based_on_R-Code_Tobias_Bluhmki”.

A version of the code and data supplement that includes the intermediate results is available on Zenodo via the following two links:

- https://zenodo.org/records/16902744?token=eyJhbGciOiJIUzUxMiJ9.eyJpZCI6Ijc5NjE0ZjQ1LTJlMzItNGY4Zi04NzhlLWIwNmRjMWUyYTI1NCIsImRhdGEiOnt9LCJyYW5kb20iOiJjYzNiYTgyZTU3OWVlNzhlMmJkZTAwYzE3YmNlOTAyMCJ9.VXbNWqJFIMnvzvuS7bHjIT26Gi1ZI5Z3NmprtF0KVMCRb7mhIGOcepLmOLTvf7uxIstdm8EYoVtRCO6uV25r9A
- https://zenodo.org/records/16903759?token=eyJhbGciOiJIUzUxMiJ9.eyJpZCI6Ijc4ZDcyN2I3LWE4MzItNDA1Mi04MTgzLWFiYWVmMGNkYTUyYyIsImRhdGEiOnt9LCJyYW5kb20iOiI3MDhkOTgyMmEzYjFiMmRhYzAwMWIwNzJlZjliOWY2NSJ9.HlZMCErtQMGKUsY6YrzHTbcqm9gCwXmo-qxWtucg-zflKCBSjgvB7JLBCpIwYNfo1RFZRJ7SlJpAhCXYoekUVA

Folder structure:

- readme.pdf
- runme.R
- /Results
    - Example_figure_2.jpeg
    - Table_3.RData
    - Table_4.RData
    - Table_5.RData
    - Table_6.RData
- /Pseudo_data
    - pseudo_data_illDeath.RData
    - pseudo_data_prog.RData
- /Functions
    - approx_true_cgrfs_fct.R
    - bootstraps.R
    - conf_bands.R
    - conf_bands_cov_prob.R
    - conf_interv.R
    - conf_interv_cov_prob.R
    - simul_and_estimate_cgrfs.R
    - ./Confidence_band
        - cov_prob_cb.R
        - help_fct_cov_prob.R
    - ./Confidence_interval
        - cov_prob_ci.R
        - loglog_ci_efron.R
        - loglog_ci_wb.R
        - quantile_efron.R
        - quantile_wb.R
    - ./Efons_bootstrap
        - efron_AJE_illDeath.R
        - efron_AJE_prog.R
        - efron_KME_comb.R
        - help_fct_efron.R
    - ./Estimators
        - KME_prog.R
    - ./Functions_based_on_R-Code_Tobias_Bluhmki
        - loglog_cb_efron.R
        - loglog_cb_wb.R
        - wb_AJE_illDeath.R
        - wb_AJE_prog.R
        - wb_KME_comb.R
    - ./Simulation
        - progr_to_illDeath.R
        - simu_progr_six_state.R

```
> sessionInfo()

R version 4.1.2 (2021-11-01)
Platform: x86_64-pc-linux-gnu (64-bit)
Running under: Ubuntu 22.04.5 LTS

Matrix products: default
BLAS:   /usr/lib/x86_64-linux-gnu/blas/libblas.so.3.9.0
LAPACK: /usr/lib/x86_64-linux-gnu/lapack/liblapack.so.3.9.0

locale:
 [1] LC_CTYPE=en_US.UTF-8       LC_NUMERIC=C               LC_TIME=de_DE.UTF-8
LC_COLLATE=en_US.UTF-8     LC_MONETARY=de_DE.UTF-8
 [6] LC_MESSAGES=en_US.UTF-8    LC_PAPER=de_DE.UTF-8       LC_NAME=C
LC_ADDRESS=C               LC_TELEPHONE=C
[11] LC_MEASUREMENT=de_DE.UTF-8 LC_IDENTIFICATION=C

attached base packages:
[1] parallel  stats     graphics  grDevices utils     datasets  methods   base

other attached packages:
[1] data.table_1.14.4 survival_3.4-0    etm_1.1.1

loaded via a namespace (and not attached):
[1] compiler_4.1.2  Matrix_1.3-4    tools_4.1.2     Rcpp_1.1.0      splines_4.1.2
grid_4.1.2 lattice_0.20-45
```
