# Supplementary figures and images for: Non‐Markov Nonparametric Estimation of Complex Multistate Outcomes After Hematopoietic Stem Cell Transplantation
Source: Biom J. 2025 Oct 29;67(6):e70082. doi: 10.1002/bimj.70082 (PMC12569748; doi:10.1002/bimj.70082)

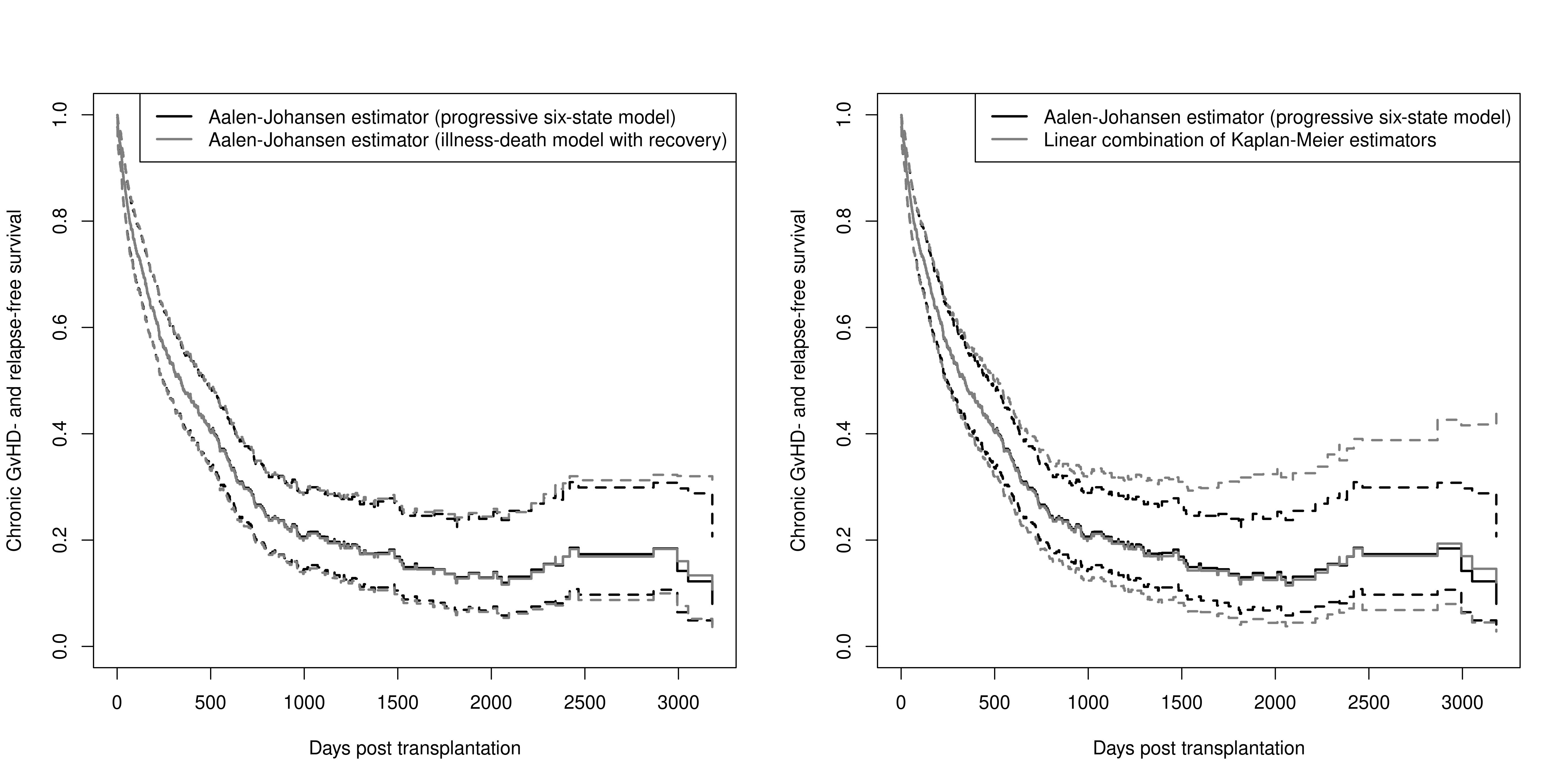

Supplement: Supplementary file 1 — Supporting File: bimj70082o‐supo‐0001o‐Code.zip. [file BIMJ-67-e70082-s001.zip › Code_supplement/Results/Example_figure_2.jpeg]
